# Dynamic Gesture Recognition Model Based on Millimeter-Wave Radar With ResNet-18 and LSTM

**DOI:** 10.3389/fnbot.2022.903197

**Published:** 2022-06-07

**Authors:** Yongqiang Zhang, Lixin Peng, Guilei Ma, Menghua Man, Shanghe Liu

**Affiliations:** ^1^National Key Laboratory on Electromagnetic Environment Effects, Army Engineering University, Shijiazhuang, China; ^2^School of Information Science and Engineering, Hebei University of Science and Technology, Shijiazhuang, China

**Keywords:** gesture recognition, millimeter-wave radar, ResNet-18, LSTM, Human-Computer Interaction

## Abstract

In this article, a multi-layer convolutional neural network (ResNet-18) and Long Short-Term Memory Networks (LSTM) model is proposed for dynamic gesture recognition. The Soli dataset is based on the dynamic gesture signals collected by millimeter-wave radar. As a gesture sensor radar, Soli radar has high positional accuracy and can recognize small movements, to achieve the ultimate goal of Human-Computer Interaction (HCI). A set of velocity-range Doppler images transformed from the original signal is used as the input of the model. Especially, ResNet-18 is used to extract deeper spatial features and solve the problem of gradient extinction or gradient explosion. LSTM is used to extract temporal features and solve the problem of long-time dependence. The model was implemented on the Soli dataset for the dynamic gesture recognition experiment, where the accuracy of gesture recognition obtained 92.55%. Finally, compare the model with the traditional methods. The result shows that the model proposed in this paper achieves higher accuracy in dynamic gesture recognition. The validity of the model is verified by experiments.

## 1. Introduction

With the rapid development of computers, there is a growing need for new ways to interact with the computer. Gesture interaction is considered a new way of HCI and becomes more and more popular. Gesture recognition technology enables the computer to understand human instructions without hardware interaction to achieve the purpose of HCI. Gestures as an intrinsic part of human communication, either complementing spoken language or replacing it altogether (Hewes, 1992). A gesture can be defined as a deliberate set of motions executed with any body part to convey a message or evoke an action (McNeill and Levy, 1982; Kendon, 1990). Gestures can be divided into two categories based on behavior: static gestures and dynamic gestures. Static gestures refer to stable shapes made by the user at a certain point in time, while dynamic gestures are a series of coherent actions produced by the user within a certain period of time, adding temporal information and action features. Recently, hand gesture recognition as a new form of HCI has become an active field of research.

On the one hand, gesture recognition technology can provide a convenient and fast way of HCI. On the other hand, it also reduces the direct contact between people and devices. In recent years, the COVID-19 pandemic has highlighted the further benefits of contactless interfaces for preventing the spread of the virus. Contactless gesture recognition can be implemented in a variety of devices, such as optical cameras and radar. Camera-based methods are more common. Gestures are captured by the camera sensor, and the gesture signals in the form of video or images are obtained, and then these signals are input into the classifier for classification (Rogez et al., 2015). However, camera-based methods have certain limitations, such as being easily affected by light and dust, the privacy protection of users is not in place, and it is easy to be attacked. Radar sensors become a major research area. The radar sensor has the characteristics of small size, easy integration, and high spatial resolution. Additionally, it can be applied to wearable devices such as mobile phones, watches, and smart headphones. In addition, it provides a better privacy protection system than visual sensors (Ahuja et al., 2021). At present, most of the radars used in radar-based gesture recognition are millimeter-wave radars. This kind of radar offers unique advantages because it works 24/7. In addition, the radar signal can capture motion even with very small amplitude changes and can accurately distinguish subtle gestures.

Considering the need for accurate classification of millimeter-wave radar gesture data, researchers have proposed several approaches to address this problem. One of the most popular and traditional classification methods for gesture recognition is the Dynamic Time Warping (DTW) method. When processing radar data with DTW, a template set needs to be constructed first, and then the classification result is obtained by comparing the difference between the template and the test data. In a previous study, researchers used the DTW method (Zhou et al., 2017) to classify 10 categories of gesture signals, and the recognition accuracy reached more than 91%. However, the DTW algorithm has the disadvantages of high computational complexity and low stability. In order to overcome these deficiencies, researchers have tried to use machine learning methods for millimeter-wave radar gesture recognition. Among many methods, popular machine learning algorithm includes Support Vector Machines (SVM), Hidden Markov Models (HMM), and K-Nearest Neighbor algorithms (KNN). By extracting the micro-Doppler features from the time-frequency map of the gesture signal, and using the SVM algorithm to classify 4 categories of gestures, an accuracy of 88.56% is obtained (Zhang et al., 2016). When using HMM to recognize 6 categories of millimeter-wave radar gestures, an HMM model is established for each gesture, and calculate the probability of each gesture through the established model. The gesture with the highest probability is the obtained classification result, and the accuracy rate reaches 88.3% (Malysa et al., 2016). But when the task of multi-class gesture recognition is performed, the HMM is not applicable. The KNN algorithm was also applied to recognize dynamic gestures (Xian et al., 2018). Compared with the traditional method, the accuracy of the KNN method is improved, but it is only for small sample datasets. Among the several machine learning methods mentioned above, manual feature extraction is required, which is not efficient. In order to overcome the difficulty that machine learning requires manual feature extraction, the deep learning algorithm for radar gesture recognition is becoming a hot field. The advantage of deep learning is that it can achieve end-to-end training, eliminating the need for machine learning algorithms to manually extract features, and simplifying the tedious step of the workload. The algorithms of deep learning in radar gesture recognition mainly include Convolutional Neural Networks (CNN) and Recurrent Neural Networks (RNN). A study uses a data fusion approach for driving gesture action recognition using 3D CNN (Molchanov et al., 2015). The Soli team used an end-to-end recurrent neural network method to recognize 4 categories of gestures through the range-Doppler feature images of radar gesture signals and obtained an accuracy of 92.1%. When using 11 categories of gestures, an accuracy of 87% is obtained (Hazra and Santra, 2018).

A classification model for 11 categories of gestures is proposed in this article, which innovatively combines two deep learning algorithms. Using the Soli dataset and the model uses ResNet-18 as the feature extractor to extract the distance-doppler features of each frame. Then extract frame-to-frame temporal features using LSTM, and finally, classify gestures through a softmax layer. Here, the input data were a group of Range Doppler Map (RDM) images which include distance and speed information for gestures. The classification accuracy of the model is 92.55%, which is higher than the previous models.

## 2. Dataset Description

Previous gesture capture devices include optical-based gesture capture devices (Yao and Li, 2015) and depth camera-based gesture capture devices (Ren et al., 2011). These devices are either susceptible to light conditions or are inconvenient to use on a daily basis. In this case, these devices are not conducive for people to use for HCI anytime and anywhere. By contrast, the advantages of millimeter-wave radar are reflected. The radar sensor can provide more and richer Doppler information without being affected by lighting conditions, and a variety of similar gestures can also produce distinguishable Doppler features. Embedding radar into electronic devices such as smartphones or watches enables the fine-grained perception of human interactions (Lien et al., 2016). By accepting a hand gesture as an input modality, electronic devices can be controlled without touching a button, boosting the reliability of the device and design flexibility. Moreover, it can heighten convenience for users. With the development of technology, a hot direction of HCI is gesture interaction using millimeter-wave radar sensors. Google's Soli project is an example of gesture recognition. Soli radar sensors can be embedded into mobile and wearable devices for interactive gesture recognition.

This article starts with Google's Soli project (Lien et al., 2016). Soli is a new sensing technology that uses miniature radar to detect gestures in the air. This specially designed radar sensor can track target movement with sub-millimeter accuracy and then process the radar signal into a series of universal interactive gestures to facilitate the control of various wearable and microdevices. Google's Soli project uses a new 60GHz Frequency Modulated Continuous Wave (FMCW) millimeter-wave radar to create a radar-based sensor optimized for HCI.

**Figure 1:**
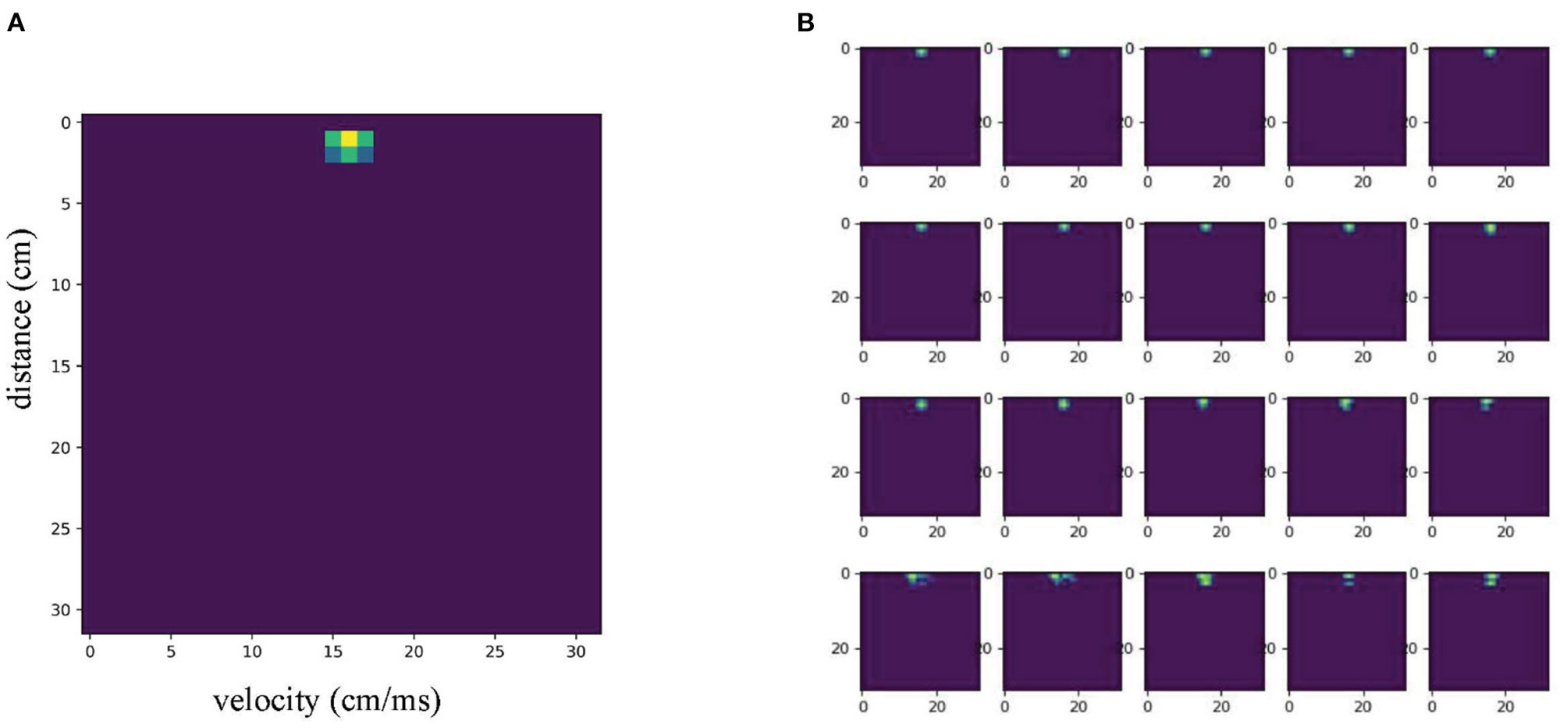
**(A)** An random example of the dataset, **(B)** 20 frames of selected images for this example.

The Soli dataset contains many groups of gesture data, each consisting of a series of 32*32 RDM images. Each group of images selects 20 frames as a sequence to represent a complete gesture. The dataset contains 11 gesture categories, from 10 different users. Each category of gesture contains 25 samples, for a total of 2,750 samples. The dataset is divided into a training set and validation set according to the ratio of 6:4. Figure 1 shows an image from a sample. Each RDM image frame is preprocessed by a per-pixel Gaussian model to remove background noise and normalize the signal to adjust the variance caused by radar reflection. Each RDM image in the Soli dataset also contains four channels of data captured by four receivers on the radar.

## 3. Methods

**Figure 2:**
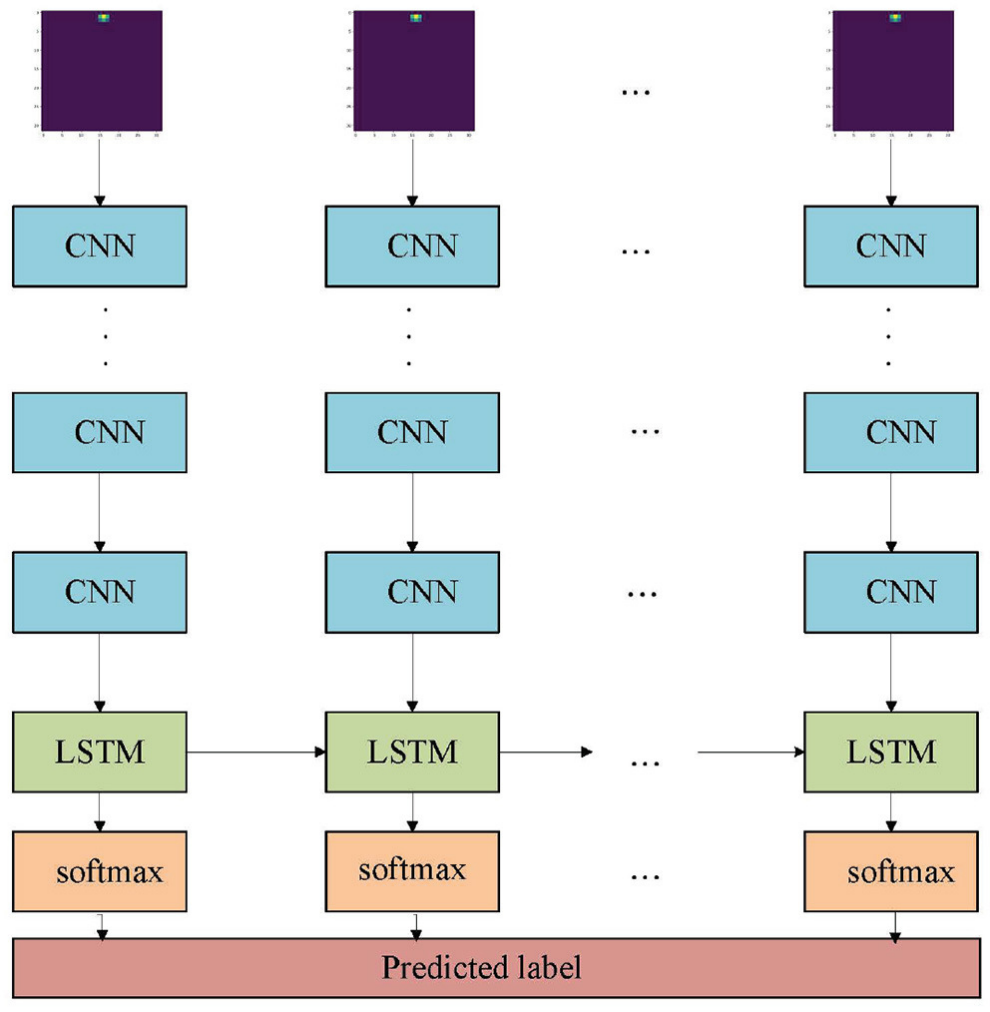
A diagram of the model architecture. The CNNs form a ResNet-18 model (He et al., 2016). The Long short-term memory (LSTM) is an RNN model with two hidden layers, each with 512 units.

A lot of existed gesture classification models are based on images (Wu et al., 2012) or videos (Cardona et al., 2019), and they rely on spatial information. The radar data does not directly contain information about shape, so several existing algorithms (Pisharady and Saerbeck, 2015) are rarely applicable. The traditional radar-based gesture recognition method can be summarized in three steps: signal transformation, feature extraction, and classification. The ResNet-18 and LSTM architectures used to build an end-to-end learning model are shown in Figure 2. The model combines the steps of signal transformation and feature extraction. ResNet-18 is used for feature extraction, and LSTM is used for temporal feature extraction. Compare the proposed model with machine learning models and deep learning models. These models included random forests (RF) (Camgöz et al., 2014) and CNN+LSTM (Wang et al., 2016).

### 3.1. ResNet-18

Deep Convolutional Neural Network (DCNN) is widely used in data classification and has made rapid progress, such as speech recognition (Dahl et al., 2011), text classification (Deng et al., 2021), video classification (Akilan et al., 2019), and image classification (He et al., 2016). The advantages of DCNN are reflected in three aspects: local area perception, sampling in space or time, and weight shared. Meanwhile, DCNN also has some disadvantages. Generally, the identification effect of shallow network layers is poor. With the increase of network layers, the identification effect is good at the beginning and then decreases. This is because the gradient disappears or the gradient explodes as the number of network layers deepens.

**Figure 3:**
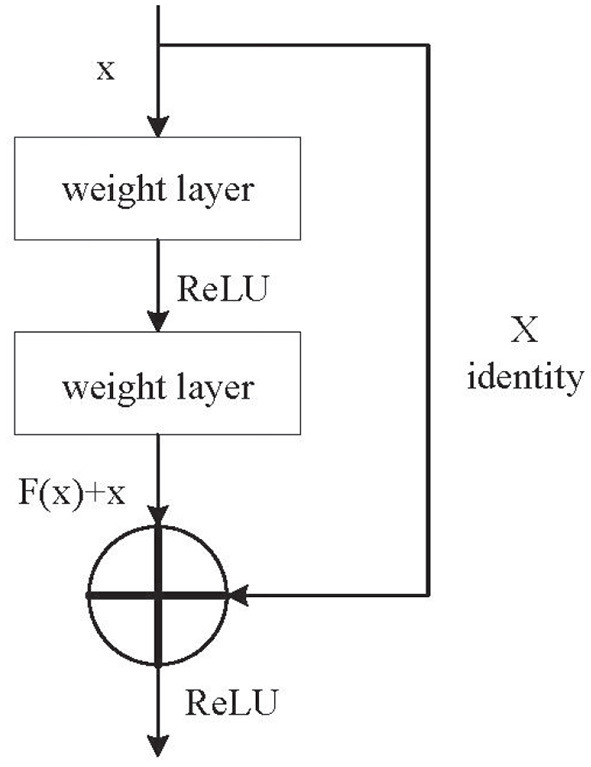
The residual block structure of ResNet.

In order to solve the shortcomings of DCNN, the researchers proposed an improved method: ResNet. The residual block was introduced into DCNN as an improvement. Figure 3 shows the structure of the residual block. Its main characteristic is the shortcut residual connection between continuous convolution layers. Gradients can flow directly through these connections, which makes training DCNN much easier by reducing the vanishing gradient effect. In Figure 3, the weight layer is the convolutional layer, *x* is the input, *H*(*x*) is the output, *F*(*x*) is the residual mapping function, and *H*(*x*) = *F*(*x*) + *x*.

The ResNet-18 was used to extract spatial features from the input data. Before classifying gestures as a sequence, each 32*32*20 image was put into a 2D CNN to extract features associated with gestures. ResNet-18 is a deep convolutional network with residual blocks. In DCNN, ResNet-18 is the relatively deep model with 11 network layers the first 9 layers are convolution layers and followed by a GAP layer that averages the sequence processing. For the simple reason that ResNet-18 here is used for feature extraction rather than classification directly, the last two layers of the ResNet-18 (the fully connected layer and the softmax layer) were removed. The activation function for the final layer was a rectified linear unit [*ReLU*(*x*) = *max*(0, *x*)]. The resulting output feature map size was 1 × 1 × 512 for each frame, which was then flattened. The feature output of size 20 × 512 for each sample is used as the feature input of the LSTM.

### 3.2. Long Short-Term Memory Networks

Recently, RNNs have been outperformed in model dynamic processes. It has been shown that the LSTM is good at processing sequence data and can mine timing information in the data (Hochreiter and Schmidhuber, 1997), as well as advantages in training longer sequence data, making it a suitable choice for dynamic gesture recognition. In this article, the radar-based gesture recognition problem is a time series problem which means that the value of a certain moment is affected by the previous moment or several moments. So LSTM is a suitable choice for dynamic gesture recognition. **Figure 5** shows the classification process.

**Figure 4:**
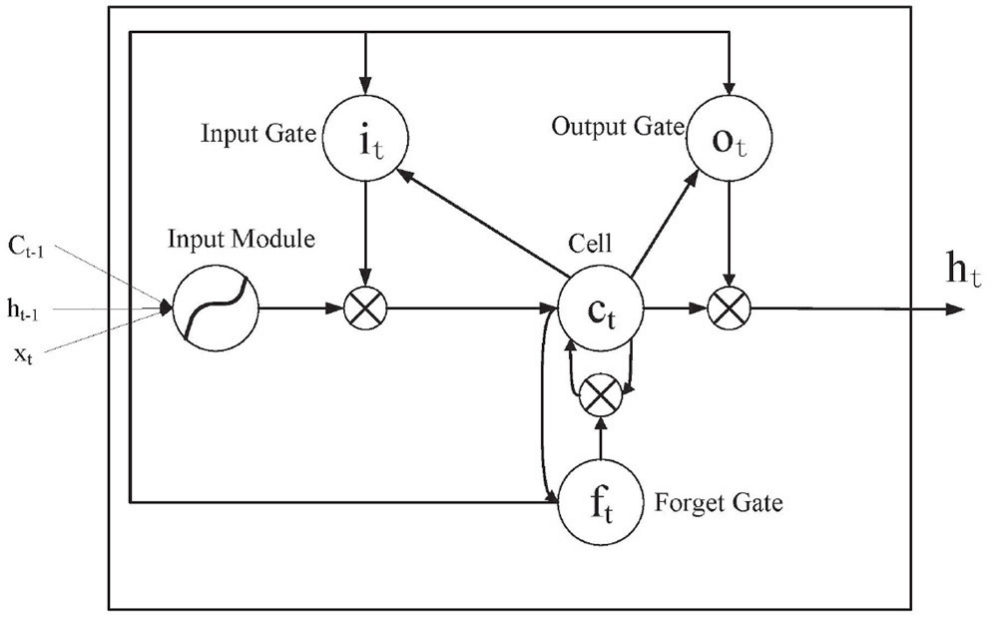
Long short-term memory internal structure diagram.

Long short-term memory is derived from the RNN and is an extension of RNN. In order to improve the accuracy, the traditional RNN usually adopts the method of deepening the number of layers. However, the traditional RNN may have the problem of gradient disappearance or explosion as the number of layers deepens. To alleviate this problem, the gate function is introduced by LSTM. It is used to store, modify, and access the internal state. A general LSTM cell is composed of the input, forget, and output gate (Refer to Figure 4 for an illustration). In Figure 4, *x*_*t*_ is the input sequence element value at time *t*. *i* is the input gate that determines how much information *x*_*t*_ currently reserves or does not reserve for the current state *c*_*t*_. *c* is the cellular state or memory unit, that controls the transmission and is at the heart of the network. *f* is the forget gate, which determines how much of the cell state *c*_*t*−1_ from the previous moment is saved to the current *c*. *o* is the output gate, which determines how much *c*_*t*_ passes to the output *h*_*t*_ in the current state. *h*_*t*−1_ refers to the state of the hidden layer at time *t* − 1.

In Figure 4 *x*_*t*_ is the feature vector extracted for each cell, the relation between input, internal state, and output is formulated as follows:


(1) $$\begin{array}{l} i_{t}=\sigma \left(W_{xi}x_{t}+W_{hi}h_{t-1}+b_{j}\right) \end{array}$$

(2) $$\begin{array}{l} f_{t}=\sigma \left(W_{xf}x_{t}+W_{hf}h_{t-1}+b_{f}\right) \end{array}$$

(3) $$\begin{array}{l} i_{t}=\sigma \left(W_{xo}x_{t}+W_{ho}h_{t-1}+b_{o}\right) \end{array}$$

σ is the logistic sigmoid function, is given by $\sigma \left(x\right)=\frac{e^{x}}{e^{x}+1}$. The input gate, forget gate, output gate, and cell activation are respectively represented by *i, f, o*, and *c*, as Figure 4 shows. The results of *i*_*t*_, *f*_*t*_, *o*_*t*_ are the current input sequence *x*_*t*_ and the previous state output *h*_*t*−1_ multiplied by the corresponding weight plus the corresponding bias and finally obtained by the sigmoid activation function, the cell and hidden states are computed as follows equations (Hochreiter and Schmidhuber, 1997):


(4) $$\begin{array}{l} \bar{c_{t}}=tanh\left(W_{xc}x_{t}+W_{hc}h_{t-1}+b_{c}\right) \end{array}$$

In Equation (4), the instant state $\bar{c_{t}}$ of the current moment unit is activated using the *tanh* activation function. Additionally, the new cell state *c*_*t*_ is a combination of current memory $\bar{c_{t}}$ and long-term memory *c*_*t*_ − 1.


(5) $$\begin{array}{l} c_{t}=f_{t}\cdot c_{t-1}+i_{t}\cdot \bar{c_{t}} \end{array}$$

The output *h*_*t*_ of the LSTM unit can be calculated by Equation (6)


(6) $$\begin{array}{l} h_{t}=o_{t}\cdot tanh\left(c_{t}\right) \end{array}$$

A series of learned parameters form the weight *W*. In the above formula, *W*_*xi*_, *W*_*xf*_, *W*_*xo*_, and *W*_*xc*_ are the weight vector of the input layer to input gate, forget gate, output gate, and cell state. *W*_*hi*_, *W*_*ho*_, *W*_*hf*_, and *W*_*hc*_ are weight vectors of hidden layer to input gate, output gate, forget gate, and cell state. *b*_*i*_, *b*_*o*_, *b*_*f*_, and *b*_*c*_ are biased for input gate, output gate, forget gate, and cell state. *tanh* is a hyperbolic tangent activation function, is given by $tanh\left(x\right)=\frac{\left(e^{x}-e^{-x}\right)}{\left(e^{x}+e^{-x}\right)}$. The operator· represents the multiplication of vector elements.

### 3.3. ResNet-18 and LSTM

**Figure 5:**
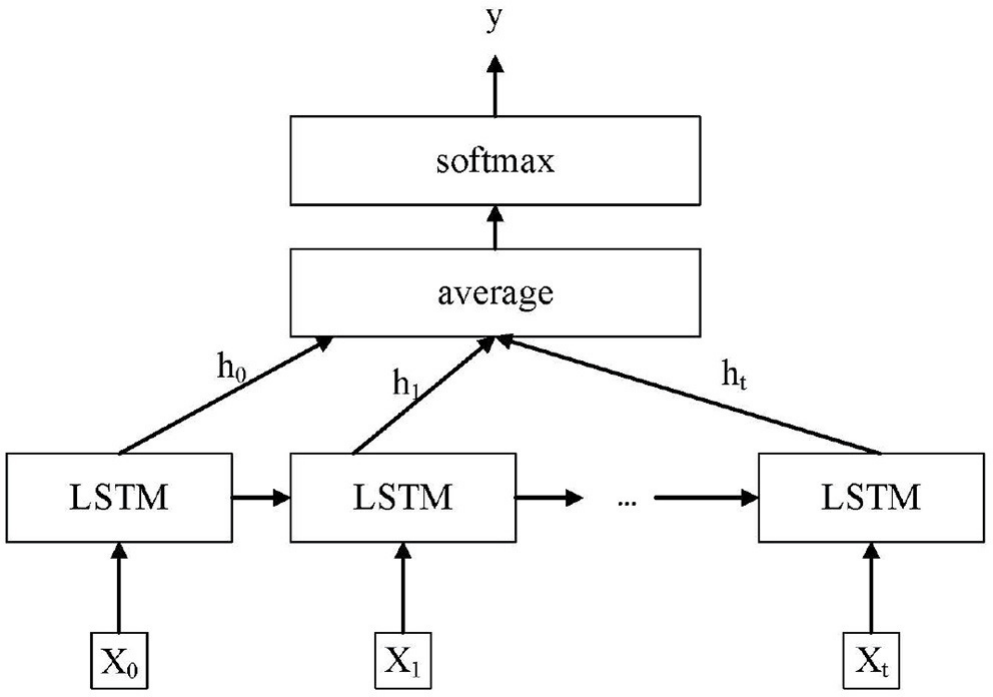
Long short-term memory classification model.

As shown in Figure 5, the ResNet-18 and LSTM model is proposed in this article for dynamic gesture recognition. Compared to traditional CNN, ResNet-18 reduces training error while adding more layers. This is because residual blocks have been added to the ResNet-18. In residual blocks, the input can faster forward propagation across layers. The addition of residual blocks solves the problem of gradient disappearance with the deepening of network layers. Besides, batch normalization was used to improve numerical stability and make the training model easier. This article adopts ResNet-18 to extract spatial features of dynamic gestures. Then the extracted features are transmitted to LSTM, and further temporal feature extraction is carried out on the sequence data through LSTM to solve the long-term and short-term dependencies between the data. LSTM makes it easier to learn long-term dependence. This is because compared to traditional RNN, LSTM introduces the idea of self-circulation. One of the key extensions is that the weight of the self-circulation is context-dependent, rather than fixed. LSTM can generate the path of gradient continuous flow for a long time, thus solving the problem of gradient attenuation in traditional RNN. LSTM performs well on challenging sequence processing tasks. The gesture recognition problem studied in this article is a temporal problem. So LSTM is used for feature extraction in the time domain. Finally, the module composed of a full connection layer is used to output the classification results.

These two deep learning architectures (ResNet-18 and LSTM) are trained jointly in an end-to-end way rather than individually. For training, each Range-Doppler image belonging to a gesture sequence is passed through the ResNet-18 for feature extraction. The features extracted by ResNet-18 are passed as input to the LSTM layer. LSTM is given an input sequence *x* = (*x*_0_, *x*_1_, …, *x*_*t*_) wherein our case that *x*_*t*_ is the feature vector extracted by the ResNet-18 at time *t*. After the ResNet-18, there is an LSTM layer, which is composed of 512 units. Then the LSTM output at all times is weighted and averaged as the upper representation. Finally, through a softmax layer, carry out the operation of full connection, and finally get the category ŷ of the predicted results.

### 3.4. Implementation Details

The gesture recognition problem is defined as a multi-classification problem. The experiment is divided into four stages: data processing stage, definition stage, training stage, and evaluation stage. The data processing stage includes data set division and label normalization. The definition stage includes the definition of model structure, loss function, optimizer, learning rate, and other parameters. In the training stage, use the model proposed in the article to train on the training set. In the evaluation stage, the validation set is used to evaluate and the results are obtained. The data set is divided into a training set and a test set in a ratio of 6:4. The training set includes 1,650 samples and the test set includes 1,100 samples. There are 11 categories of datasets used in this article, each sample in the data set corresponds to only one category respectively, and the label adopts one-Hot encoding, which is the representation of classification variables as binary vectors. The one-Hot encoding first requires that the classification values be mapped to integer values. Each integer value is then represented as a binary vector, which is zero except for the index of the integer, which is marked as 1. Encoding the labels makes it very convenient to calculate the loss function or accuracy. The last layer of the model is defined as a classification output layer, which enables the model to classify samples fed into the network. The softmax layer is used for multi-classification problems. The softmax function is the activation function of this layer and the categorical_crossentropy is the loss function. Two metrics are defined to evaluate the quality of the network model, accuracy, and loss. The loss function adopts the cross-entropy loss function, which is defined as follows:


(7) $$softmax(y_{i})=\frac{e^{y_{i}}}{\sum_{i=1}^{n}e^{y_{i}}}$$

(8) $$\begin{array}{l} L\left(y,ŷ\right)=-\frac{1}{n}\overset{n}{\underset{i=1}{\sum }}ŷ\times log\left[softmax\left(y_{i}\right)\right] \end{array}$$

In the above formula, *n* is the total sample number of training, *y*_*i*_ is the true value label, ŷ is the predicted label. In the loss function, it only cares about the probability of prediction for the right category. The optimization function selects Root Mean Square Prop (RMSProp) algorithm, and the formula of the algorithm in the *t* round iteration is shown below:


(9) $$\begin{array}{l} s_{dw}=\beta s_{dw}+\left(1-\beta \right)dW^{2} \end{array}$$

(10) $$\begin{array}{l} s_{db}=\beta s_{db}+\left(1-\beta \right)db^{2} \end{array}$$

(11) $$\begin{array}{l} W=W-\alpha \frac{dW}{\sqrt{s_{dW}}+\varepsilon } \end{array}$$

(12) $$\begin{array}{l} b=b-\alpha \frac{db}{\sqrt{s_{db}}+\varepsilon } \end{array}$$

RMSProp algorithm uses differential square weighted average for the gradient of weight *W* and bias *b*, which is beneficial to correct the swing amplitude of gradient, making the swing amplitude of each dimension smaller, and making the convergence of network function faster. In the above formula, *s*_*dW*_ and *s*_*db*_ are the gradient momentum accumulated by the loss function during the previous *t* − 1 iteration, respectively, ε is a number used to smooth gradients, usually to the power of 10^−8^.

Before analyzing a frame sequence of a gesture, each individual 32×32×4 frames was fed through ResNet-18 to extract relevant spatial features. The first layer of ResNet-18 is a 3 × 3 convolutional layer with 64 output channels and stride 1. The residual block first has two 3 × 3 convolutional layers of the same output channel and stride 2. Each convolutional layer is followed by a batch normalization layer and ReLU activation function layer. Besides, use an additional 1 × 1 convolution layer to modify the number of channels and stride of the convolutional layer. The ResNet-18 is composed of 4 residual blocks. Each residual block doubles the number of channels of the previous residual block. In the model proposed, in this paper, the number of output channels used is 64, 128, 256, 512. The output for each frame was 1 × 1 × 512. Then flattened each feature map to 512 × 1. In order to avoid overfitting, a dropout layer is added after the dense layer, and the probability value is set to 0.5. A feature output map of shape 20 × 512 for every 20 frames dynamic gesture to be used as input for the LSTM. The LSTM in this article is many-to-one since then fed a sequence of 20 frames into the LSTM to extract temporal features. The final size of the LSTM network was chosen to be 1 layer with 512 units. Finally, the classification of 11 categories of dynamic gestures was achieved through a fully connected layer with 11 units, and the activation function uses the softmax function.

During the experiment, the parameters used in the experiment are set as follows: the learning rate is 0.00001, the epochs are 100, and the batch_size is 16. The model proposed in this article is implemented on TensorFlow (Abadi et al., 2016), a deep learning framework. This model is built using this framework and trained the network with 100 epochs using small-batch samples. To prevent overfitting, add a dropout layer with a probability of 0.5 after the fully connected layer. Specific training parameters are shown in Table 1. The entire network is trained in an end-to-end manner. Computations are performed on Google's Colab using GPUs.

**Table 1:** Traning parameters.

**Parameters**	**Setting**
Framework	TensorFlow
Epochs	100
Loss function	Cross entropy loss
Optimizer algorithm	RMSProp
Learning rate	0.00001
LSTM	Units = 512
LSTM Activation	ReLU
Dense	Units = 11
Dense activation	softmax

## 4. Results and Discussion

**Figure 6:**
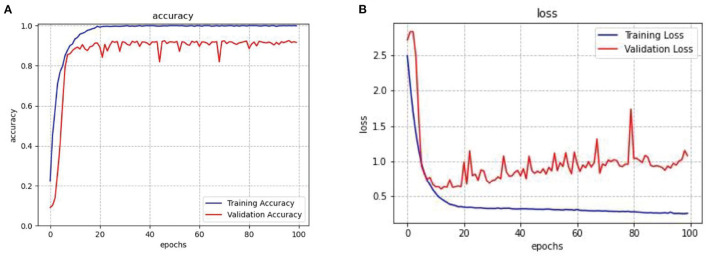
**(A)** Accuracy change in ResNet-18 and LSTM model. **(B)** Loss change in ResNet-18 and LSTM model.

After repeated training on the data, the average optimal accuracy is 100% on the training set and 92.55% on the validation set. Then conducted tests on the test set, and the result showed that the accuracy of the recognition rate was close to 93%. In order to see the experimental result more intuitively, a graph shows the change curve of the loss rate and accuracy rate of the network model with the increase of training epochs in Figure 6, the blue line represents training operation and the red line represents validation operation. The result shows that in the network model constructed in this article, convergence began after about 20 epochs, and since then the accuracy rate gradually increased while the loss rate gradually decreased. After 20 epochs, the accuracy of the training set converges to 99.95%, and that of the validation set converges to 92.55%. The results of repeated training on the model are shown in Table 2. The results show that there is little difference in the results obtained after repeated training. It shows that the model presented in this article is stable.

**Table 2:** Model training results.

**Time**	**Optimal accuracy on** **the training set (%)**	**Optimal accuracy on** **the validation set (%)**
1	100.00	92.15
2	100.00	92.73
3	100.00	92.32
4	100.00	92.64
5	100.00	92.91

**Figure 7:**
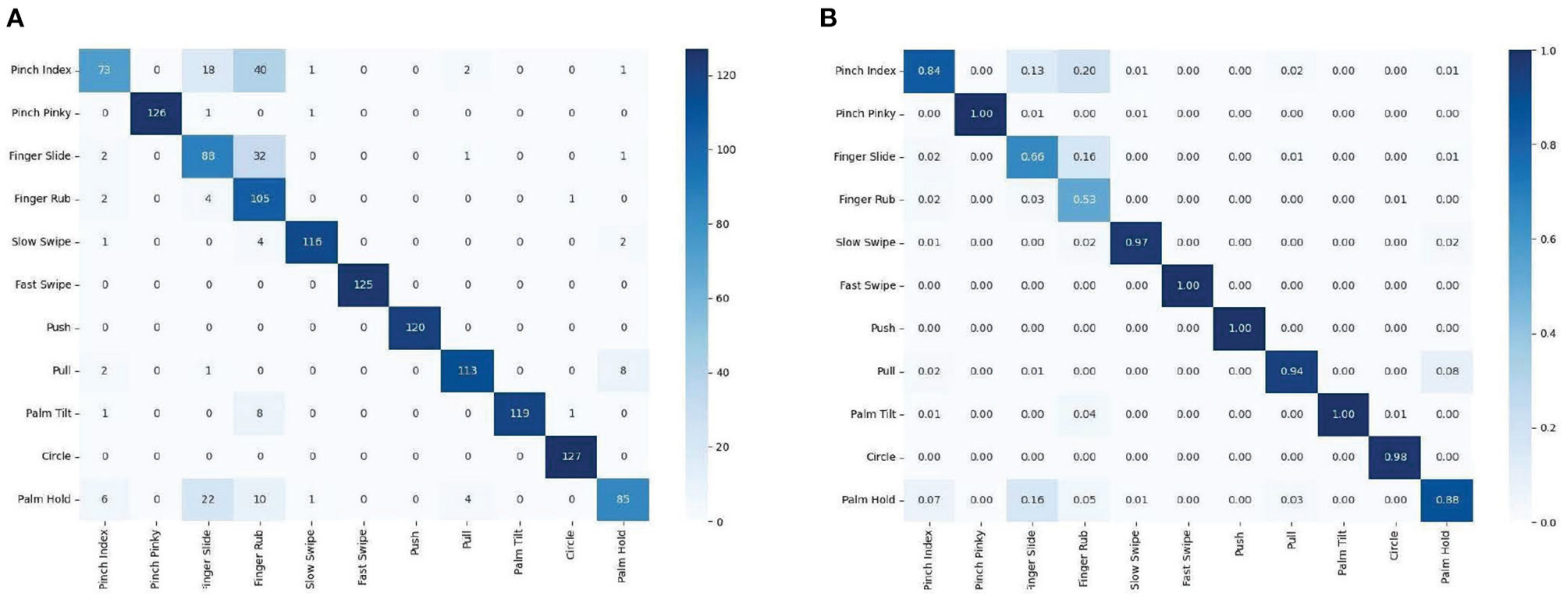
Confusion matrix for the Soli 60–40% split for training and evaluation. **(A)** Confusion matrix for 11 categories of gestures of 10 users, **(B)** Normalized to percentage confusion matrix for 11 categories of gestures of 10 users.

Then test with the trained model, use the same data set, and finally get a confusion matrix as shown in Figure 7. It demonstrates that the model proposed in this article achieves high accuracy in the classification of 11 categories of gestures, has superiority in classification task of multi-class gesture.

This article demonstrates the ResNet-18 and LSTM classification model for radar-based signals to classify 11 categories of gestures. The model can classify up to 11 different millimeter-wave radar hand signals with an average accuracy of 92.55%. The result shows that the model proposed in this article can also accurately distinguish gestures with small differences. This allows the model to be easily generalized to a smaller variety of gesture datasets. Finally, the model was compared with the other two models: CNN+LSTM and RF. The result shows that our model is better than the other radar-based solutions reported in the literature (demonstrated in Table 3). The RF method classifies only 4 categories of gestures, while the model proposed in this article classifies 11 categories of gestures and achieves higher accuracy. The comparison shows the superiority of ResNet-18 and LSTM.

**Table 3:** State-of-the-art radar-based gesture recognition.

**Network**	**Best accuracy (%)**	**Dataset**	**References**	**Gestures**
ResNet-18+LSTM	**92.55**	Soli	this model	11
CNN+LSTM	87.17	Soli	Wang et al., 2016	11
Random forest	92.1	Soli	Lien et al., 2016	4

## 5. Conclusion and Future Study

A millimeter-wave radar gesture recognition model was proposed in this article which was based on ResNet-18 and LSTM. First, the processed data set was put into the model. Second, the ResNet-18 was used to extract the spatial features of the data. Finally, the LSTM was used to extract the temporal features, and the softmax layer is used for classification. The model can recognize up to 11 different categories of gestures and achieve a high average accuracy. The result shows that the average optimal accuracy is 92.55%. By conducting experiments, the result shows that the model achieves higher accuracy than previous models due to its characteristic of extracting temporal and spatial features, which have a certain significance and value.

To sum up, the model proposed in the article is good at dealing with radar data displayed in the form of Doppler images. It shows the feasibility of this model in radar gesture recognition and has wider application potential in many fields such as pattern recognition. It can be easily generalized to other applications.

In future research, how to improve the generalization of the model is a key issue. The next step is to use millimeter-wave radar to collect multiple samples of different categories of gestures, and then create our own dataset and improve on the existing model. The ultimate goal is to achieve higher accuracy and fewer computing resources. After the above study is completed, a variety of experiments would be conducted to analyze the characteristics of the data and the model and determine which features are important for gesture recognition and which type of data is better for the model. This is especially important for more accurate gesture recognition in the future.

## Data Availability Statement

The original contributions presented in the study are included in the article/supplementary material, further inquiries can be directed to the corresponding authors.

## Author Contributions

YZ and GM: concept, theory, and resource. LP: experiment, writing, and survey. MM: software and hardware. SL: edit and review. All authors contributed to the article and approved the submitted version.

## Funding

This study was supported by a grant from the National Key Laboratory of Scientific and Technology Foundation of China (Grant no. 6142205190101), Natural Science Foundation of Hebei Province (Grant no. F2018208116), and Key Projects of Science and Technology in Higher Education Institutions of Hebei Province (Grant nos. ZD2020176 and ZD2021048).

## Conflict of Interest

The authors declare that the research was conducted in the absence of any commercial or financial relationships that could be construed as a potential conflict of interest.

## Publisher's Note

All claims expressed in this article are solely those of the authors and do not necessarily represent those of their affiliated organizations, or those of the publisher, the editors and the reviewers. Any product that may be evaluated in this article, or claim that may be made by its manufacturer, is not guaranteed or endorsed by the publisher.
